# A new device for deep cervical artificial insemination in gilts reduces the number of sperm per dose without impairing final reproductive performance

**DOI:** 10.1186/s40104-019-0313-1

**Published:** 2019-01-28

**Authors:** Pedro J. Llamas-López, Rebeca López-Úbeda, Gustavo López, Emily Antinoja, Francisco A. García-Vázquez

**Affiliations:** 1 Department of Physiology, Faculty of Veterinary, Campus Mare Nostrum, University of Murcia, 30100 Murcia, Spain; 2 Department of Cell Biology and Histology, Faculty of Medicine, Campus Mare Nostrum, University of Murcia, 30100 Murcia, Spain; 3 Institute for Biomedical Research of Murcia (IMIB-Arrixaca), Murcia, Spain; 4 Cherkizovo Group, Lipetsk, Russia

**Keywords:** Cervix, Intrauterine, Nulliparous, Porcine, Post-cervical insemination

## Abstract

**Background:**

The aim of this study was to evaluate the reproductive performance of a new artificial insemination (AI) device specifically designed for gilts (Deep cervical AI, Dp-CAI) by means of which the sperm is deposited deeply in the cervix (8 cm more cranial than in traditional cervical insemination-CAI). New AI techniques have arisen in recent decades in the porcine industry, such as post-cervical artificial insemination (PCAI), which involves depositing the sperm in the body of the uterus [through a catheter (outer tube)-cannula (inner tube)] rather than by CAI. Although the PCAI method has been successfully applied in farm conditions to reduce sperm doses without impairing the reproductive performance, this technique has limitations in gilts mainly because of the difficulty involved in introducing the inner cannula through the cranial part of the cervix. For this reason, the Dp-CAI method described herein may be considered as an alternative to CAI and PCAI methods in gilts.

**Results:**

Gilts were divided in two experimental groups: 1) Dp-CAI: gilts (*n* = 1166) inseminated using 1.5 × 10^9^ sperm/45 mL; 2) CAI (as a control group): gilts (*n* = 130) inseminated using 2.5 × 10^9^ sperm/85 mL. The Dp-CAI method was successfully applied in 88.90% of the gilts, with no differences detected between gilts with 1 or 2 previous oestrus cycles, although the catheter could be introduced more deeply in 2 oestrus gilts (*P* < 0.05). As the length of the insemination device that could not be introduced increased (at the moment of insemination), so the success rate of the Dp-CAI device fell, as did the total number of piglets born. When the reproductive output in CAI and Dp-CAI was compared, none of the parameters analysed [pregnancy and farrowing rates (%), and number of piglets born (total and live)] showed significant differences.

**Conclusions:**

The use of the Dp-CAI technique provides a new AI method as an alternative to CAI and PCAI for pigs. The device, especially designed for gilts, was used with a high degree of success reducing conventional sperm doses without impairing reproductive parameters.

**Electronic supplementary material:**

The online version of this article (10.1186/s40104-019-0313-1) contains supplementary material, which is available to authorized users.

## Background

Nowadays, more than 85% of swine insemination in developed countries is performed using assisted reproduction techniques [1, 2]. The successful use of artificial insemination (AI) has made it possible to improve many aspects of the industry, including fertility, genetic, biosecurity and disease control, and labour and production efficiency (reviewed by [3]). However, it is necessary to continue developing new strategies for use under field conditions in order to improve efficiency in swine AI. One of the strategies would be to optimize the number of females inseminated with each collected ejaculate by reducing the number of sperm used per dose [4–6]. At present, the two methods available to reduce the sperm cells per dose used in traditional AI (cervical AI-CAI), are post-cervical insemination (PCAI) [7, 8] and deep intrauterine insemination (DIUI) [9]. Of these, PCAI is considered the best alternative in farm conditions due to its simplicity, effectiveness and reproductive performance [7, 8]. This method consists of depositing semen in the uterine body [through special device consisting of a cannula (inner tube) inserted in a catheter (outer tube)] [10] before the uterine horn bifurcation thus avoiding the cervical barrier to sperm transport [11]. By contrast, in the CAI method, sperm deposition occurs directly in the cervix. Besides reducing the number of spermatozoa used per dose without impairing reproductive performance (2–3 fold less sperm per dose compare to CAI; [8]), PCAI offers several advantages, of particular note being the increased number of sperm doses produced per boar (reducing the number of boars needed per farm) [12], faster genetic improvement and labour efficiency [3], all of which result in substantial economic benefits [8, 13].

However, the use of PCAI has certain limitations that need to be considered. While the success rate of introducing the insemination device in multiparous sows is higher than 95% (75% at the first attempt) [7, 11, 14, 15], this is not the case with young females, whether nulliparous or primiparous. First reports described a medium to high level of difficulty in inserting the PCAI device into the uterus of 30% to 45% of primiparous females [15], being impossible in ~ 13% of cases [16]. In the case of nulliparous females, full success was attained only in ~ 20% of cases using a PCAI multiparous device, although this low success rate increased to ~ 60% at the first attempt using a catheter specifically designed for PCAI in nulliparous females [17], still far from the success rate in multiparous sows.

The fact that PCAI cannot be performed consistently in young females could be due to the smaller dimension of the cervix, which would become the major barrier because the reproductive tract would not have undergone total physical development [18]. With each parturition, the cervix of a sow becomes more relaxed, facilitating passage of the inner cannula through the cervical canal [19].

Precisely, one of the critical points in the porcine breeding system is the high percentage (~ 50%) of replacement among multiparous sows on pig farms [20, 21], which makes nulliparous and primiparous females the largest farrowing group on these farms [22]. Further, the reproductive performance of gilts is essential for success swine breeding programmes and production systems. Therefore, any effort to optimize productive potential of this population of females could have an important benefit for the industry.

For all the reasons mentioned above, the aim of this study was to test a new AI device, specifically designed for use in nulliparous females, taking into account their anatomical limitations and allowing the deposition of semen deep in the cervix. The study first evaluated the degree of success of the new device (Deep-Cervical Artificial Insemination, Dp-CAI) in nulliparous females, and then the reproductive performance (pregnancy and farrowing rates, and litter size) obtained with the new AI method compared with CAI, but reducing the sperm number per insemination (CAI: 2.5 × 10^9^ sperm/85 mL; Dp-CAI: 1.5 × 10^9^ sperm/45 mL). In both studies, the number of previous oestrus cycles and the depth that the insemination device could be inserted (measured as the length of the device protruding from the gilt during AI) were related with the level of success and the reproductive performance attainable with the new device.

## Methods

### Animals

The study was carried out under field conditions from March to July 2016 (Cherkizovo Group). The gilts used were F1 (Landrace Sire x Large White Dam) all sourced from the same farm. Boars (337 PIC and 1–4 years old) of proven fertility were used in this study. Gilts were fed ad libitum until insemination, while boars and inseminated gilts had a restricted feeding regime according to their nutritional requirements. Water was available ad libitum. Temperature levels were controlled automatically by a climate control system which maintained the temperature at 18–22 °C.

### Sperm collection

The semen used for AI was delivered daily from a commercial boar stud belonging to the same company. Each boar was used to obtain semen every 5 d by a trained worker using gloves and a collection bag with filter (Blue Bag, Minitube). The semen ejaculate was analysed using a computer-assisted spermatozoa motility analyzer (CASA) (AndroVision® system, Minitube) for spermatozoa motility, morphology and concentration. For the study, only boars with > 85% spermatozoa progressive motility and < 20% abnormal spermatozoa morphology were used. Ejaculates which met the minimum criteria were further diluted with commercial extender (Androstar®, Minitube) and then pooled into groups of three before being stored. Semen doses were prepared in 85 and 45 mL bottles containing 2.5 × 10^9^ and 1.5 × 10^9^ spermatozoa, respectively. Semen doses were conserved at 16–18 °C and used for AI within 48 h after collection. Sperm doses from the same boar were divided in the same proportion among experimental groups to avoid any boar effect.

### Artificial insemination assays

Exposure to boars to detect estrus was initiated at 25 weeks of age, allowing each gilt 10 min of nose to nose contact with a vasectomized mature boar while experienced workers applied back pressure. Standing reflex, vulva swelling, and redness were considered signs of estrus and were recorded. The criterion for insemination was a minimum of one oestrus cycle with any previous service. Each gilt received two AI sessions, the first on the day estrus was detected and the second, 24 h later. Females were weighed before insemination in order to ensure similar weights in all the experimental groups. The AI procedure was performed in individual crates.

**Fig. 1:**
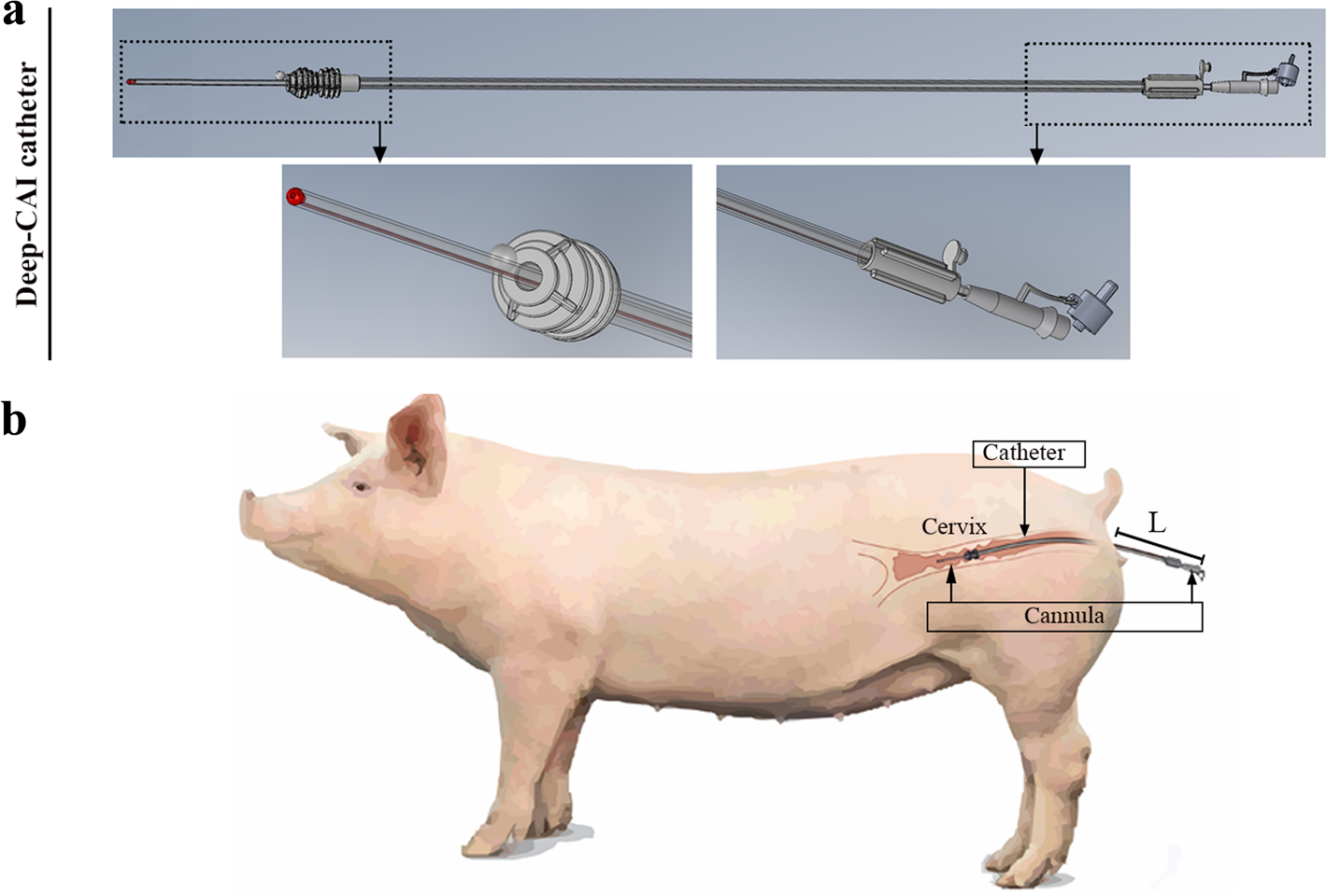
**a** Device designed for deep cervical artificial insemination (Dp-CAI) in gilts. The main characteristics of the device are as follows: 1) catheter: 52 cm in length with 17 mm diameter multiring tip; 2) inner cannula: 65 cm in length (reaching 8 cm further than the catheter) and 3 mm in diameter. **b** Representative image showing the new AI device placed in the uterus of a gilt. Semen deposition occurs deep in the cervix. “L” in the image represents the length (cm) of the device protruding from the gilt during insemination (from the vulva to the end of the insemination device)

Deep cervical AI (Dp-CAI) was performed using a new kit composed of a device which combined catheter and cannula (Fig. 1) (manufactured by Import-vet, S.A., Barcelona, Spain), which consists of a 65-cm flexible cannula (60 cm of tube + 5 cm of rear part) of 3 mm diameter (in contrast to the 3.5 mm commonly used in PCAI devices) inserted into a conventional cervical catheter (52 cm length) with multiring tip (17 mm diameter, instead of the 22 mm used in PCAI devices). From the front end of the cervical catheter to the end of the inner cannula there is a distance of 8 cm (compared with the 16 cm used for PCAI). The tip of the inner cannula ends with a rounded plastic plug with holes to permit the passage of the sperm dose. The device was designed after a study of the genital tract of nulliparous females (50.39 cm from the vulva to the end of the cervix) [18], thus ensuring intracervical deposition after Dp-CAI. Before the Dp-CAI device was inserted, the tip end of the catheter was lubricated with a non-spermicidal and sterile gel. The multiring tip was inserted through the vagina into the cervix. Then, the inner cannula was inserted through the catheter. The boar was not present at the moment of AI (unlike during CAI), thus avoiding closing of the cervix and facilitating cannula penetration. Each gilt received 1 dose per AI of 45 mL containing 1.5 × 10^9^ spermatozoa. As in CAI, the insemination dose was introduced slowly to reduce backflow because the deposition is still in the cervix but deeper. Moreover, during Dp-CAI the rear part of the cannula is attached to the back of the catheter (Fig. 1a) to prevent backflow during insemination through the AI device itself. Just after insemination, the inner cannula was removed, and the catheter was left inside the cervix 10–15 min before being removed.

Cervical AI (CAI) was performed with the same device used for Dp-CAI but without the inner cannula (using only the catheter). Gilts were inseminated with the boar present and using a non-spermicidal gel to introduce the catheter. An experienced worker applied back pressure while the semen flowed into the uterus. Each gilt received 1 dose per AI of 85 mL containing 2.5 × 10^9^ of spermatozoa. The insemination dose was introduced slowly into the female genital tract. After insemination, the catheter was left inside the cervix 10–15 min before being removed.

### Return to estrus and pregnancy diagnosis

Gilts were checked for estrus 21 d after insemination by boar exposure but with no contact, while an experienced worker applied back pressure in search of a standing reflex. Any gilt which showed estrus signs was taken out and re-allocated to another pen. Pregnancy was confirmed 35 d after AI by transabdominal ultrasonography (MS Multiscan Digital, 5 MHz sector probe, MS Schipper).

### Experimental design

A total number of 1296 gilts were divided into two groups: CAI (*n* = 130; average weight: 142.35 ± 8.06 kg) and Dp-CAI (*n* = 1166; average weight: 142.31 ± 8.27 kg). First, the application of the new Dp-CAI device for gilts (Fig. 1a) was analysed. During Dp-CAI, the length-L (cm) of the insemination device protruding from the gilt (used to calculate the penetration depth of the device) was measured (Fig. 1b). The gilts were classified as successfully inseminated (%) when the inner cannula was totally introduced, and no difficulty was observed at the moment of dose application. Both parameters - length of device protruded and successful application of Dp-CAI - were analysed for the whole gilt population and according to the number of previous oestrus cycles detected [1 (*n* = 121) or 2 (*n* = 1045)]. Subsequently, reproductive performance was compared in the two experimental groups: CAI (2.5 × 10^9^ sperm cells/85 mL) and Dp-CAI (1.5 × 10^9^ sperm cells/45 mL). The studied reproductive parameters were abortion %, pregnancy %, farrowing %, litter size born (total and live) and fecundity index [Farrowing rate multiplied by the average number of total piglets born per litter (total number of piglets born per 100 inseminations)]. Additionally, pregnancy %, total and live piglets born were related with the length of the device protruding from the gilt and the number of oestrus [1 (*n* = 102) or 2 (*n* = 934)] prior to Dp-CAI application. Finally, the association between length of the device (outside the gilt) and litter size (total and live) was evaluated.

### Statistical analysis

Statistical analyses were performed using IBM SPSS v.21 (SPSS, Chicago, IL, USA). Quantitative data (length of the device, litter size and fecundity index) were analysed for normality using a Kolmogorov-Smirnov test and for homogeneity of variances using the Levene test. When neither of the tests was fulfilled, the non-parametric, Mann-Whitney test was used. Pregnancy, farrowing and abortion rates were compared using Pearson *χ*^2^, and the successful application of the Dp-CAI by Fisher’s test. Dp-CAI success related to the length of the protruding insemination device was analysed by one-way ANOVA followed by a *post hoc* Tukey Test. The differences were considered statistically significant at *P* < 0.05 and to show a tendency when *P* ≥ 0.05 and ≤ 0.75. Data are expressed as the mean ± standard deviation (SD) (length, litter size and fecundity index) or percentage (pregnancy, farrowing and abortion rates, success of Dp-CAI). A regression analysis was performed to determine the relationship between the length of the device protruding from the gilt and litter size (total and live piglets born).

## Results

**Fig. 2:**
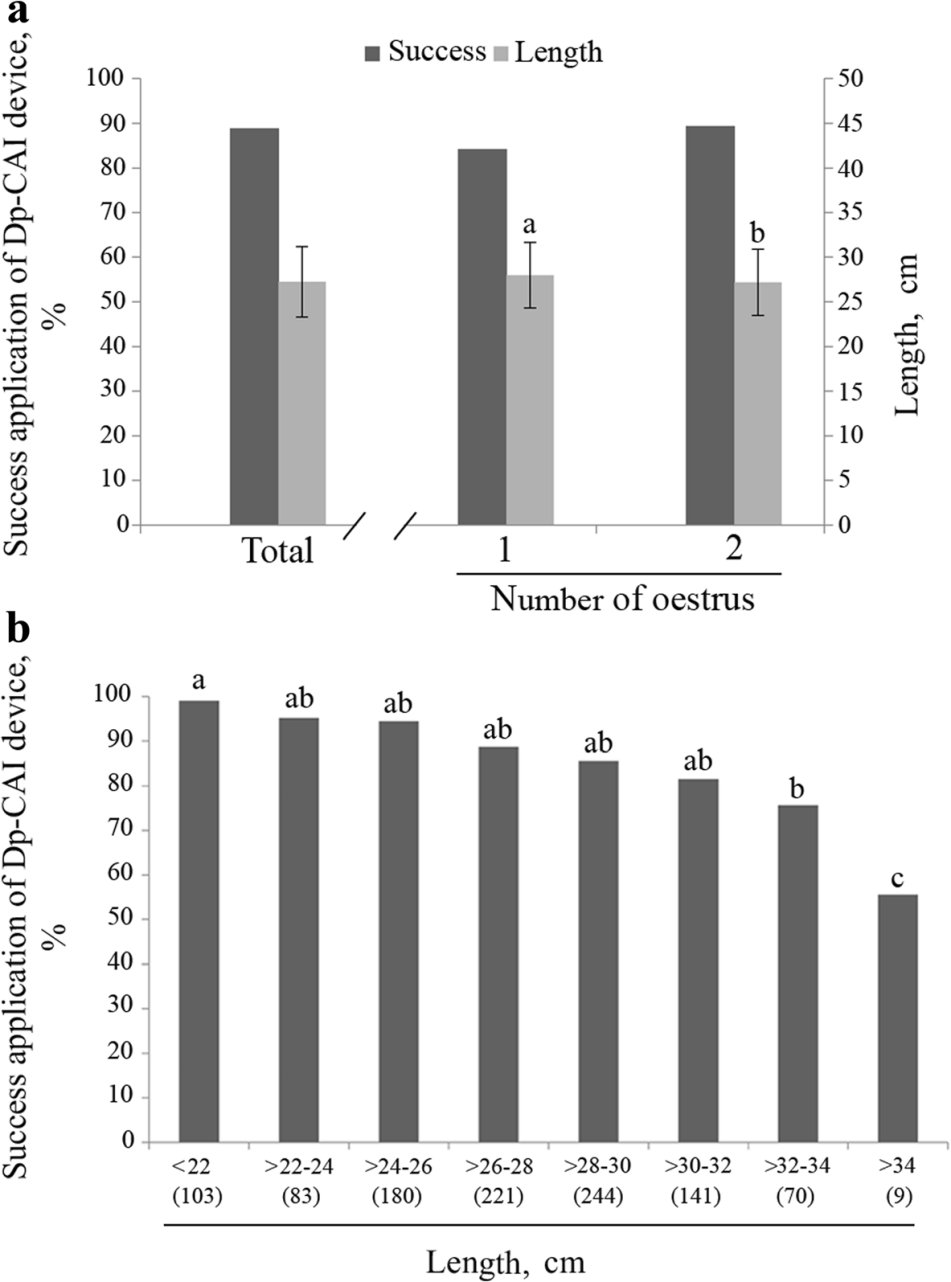
Application of deep-cervical artificial insemination (Dp-CAI) in gilts. **a** Left Y-axis represents the degree of success of Dp-CAI (dark grey bars, %); Right Y-axis represents the length (cm) of the device protruding from the gilt during AI (light grey bars, mean ± SD). Both (% of success application and length) were analysed for total number of gilt population and based on the number of previous oestrus cycles (1 or 2). Different letters (a, b) on the bars representing the number of oestrus cycles denote statistically significant differences (*P* < 0.05). **b** Success (%) of Dp-CAI application according to the length of the device protruding from the gilt during AI. Numbers between brackets indicates the n° of gilts classified in each section. Different letters (a, b, c) on the bars denote significantly different values (*P* < 0.01)

Of the total sample, 88.9% gilts were successfully inseminated by Dp-CAI using the new catheter (Fig. 2a), while it was not possible to complete the AI process in the remaining 11.1% mainly due to the difficulty of crossing the cervix with the inner cannula. When analysing the success of the technique based on the number of previous oestrus cycles (1 vs. 2), there were no significant differences although there was a tendency (*P* = 0.068) towards a higher rate in gilts that previously had 2 oestrus cycles (1-oestrus 84.3% vs. 2-oestrus 89.4% of Dp-CAI success application) (Fig. 2a). Regarding the length of the insemination device protruding from the gilt (Fig. 2a) during Dp-CAI procedure, the average length was 27.23 ± 3.7 cm. According to oestrus number, the length protruding was greater in gilts with only 1 oestrus cycle (1-oestrus 27.96 ± 3.9 vs. 2-oestrus 27.15 ± 3.7, *P* < 0.05) (Fig. 2a). When the success of the technique was analysed in detail according to the length of the device that remains outside the gilts during AI, it was observed that the success of the technique decreased as the length increased (Fig. 2b). The highest percentage of success was obtained when the length of the device remaining outside the gilt was less than 22 cm and the percentage was statistically lower when the length exceeded 32 cm (Fig. 2b).

**Fig. 3:**
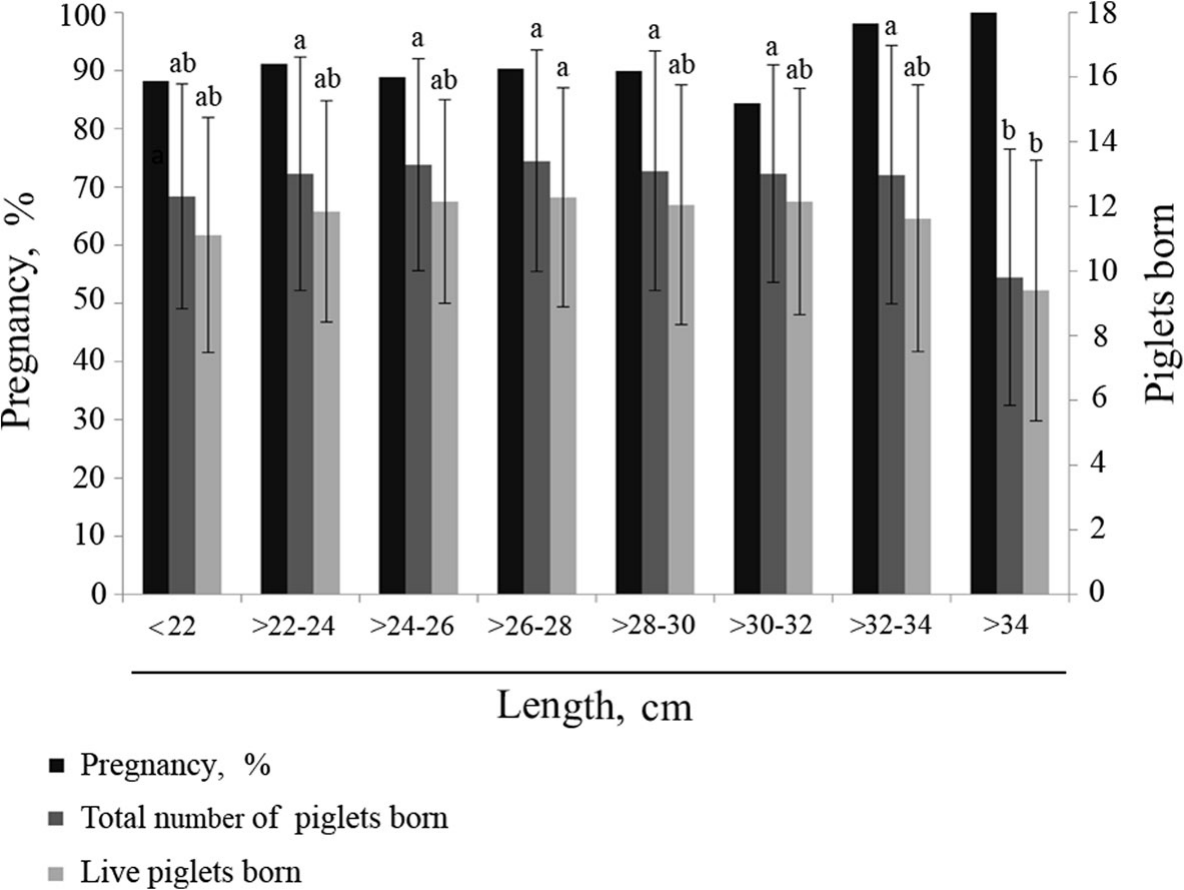
Pregnancy [Left Y-axis, represented (%) in black bars] and number of piglets born per litter [Right Y-axis, represented (mean ± SD) in dark (total) and light (live) grey bars] according to the length of the device protruding from the gilt during Dp-CAI. Different letters (a, b) on the bars of the same parameter (pregnancy, total number or live piglets born) denote significantly different values (*P* < 0.05)

When reproductive parameters were evaluated, the results showed no significant differences between the two AI methodologies used (CAI vs. Dp-CAI, *P* > 0.05) for any parameter studied (Table 1), the reproductive performance attained being similar with both methods even though 1 × 10^9^ fewer sperm per insemination were used in Dp-CAI. Moreover, when gilts inseminated by Dp-CAI were analysed according to the number of previous oestrus cycles (1 vs. 2), no differences were found in any parameter evaluated (*P* > 0.05) (Additional file 1). When the gilts were classified depending on the protruding length of the AI device during insemination (Fig. 3), the longest distance (> 34 cm) corresponded to the lowest values in terms of both total and live piglets born; however, pregnancy rates did not differ with the different lengths. Finally, a regression analysis of pooled data found no correlation between length and total number of piglets born (*y* = 0.023*x* + 12.44; *r*^2^ = 0.0006; *P* = 0.48) (Additional file 2a) or between protruding length and live piglets born when Dp-CAI was performed (*y* = 0.0381*x* + 10.929; *r*^2^ = 0.0017; *P* = 0.25) (Additional file 2b).

**Table 1:** Reproductive parameters obtained from gilts inseminated by CAI (2.5 × 10^9^ sperm/85 mL) or Dp-CAI (1.5 × 10^9^ sperm/45 mL). Data show rate (%) or mean ± SD. No statistically significant differences were observed in any of the parameters studied (*P* > 0.05)

	Number of gilts	Pregnancy, %	Farrowing, %	Abortion, %	Total born per litter	Live born per litter	Fecundity index^a^
CAI	130	87.5	83.6	3.9	13.7 ± 2.6	12.3 ± 3.2	1151.1 ± 221.3
Dp-CAI	1036	89.8	87.5	2.3	13.1 ± 3.5	12.0 ± 3.5	1147.3 ± 305.2
*P*-value		0.253	0.135	0.199	0.229	0.511	0.542

^a^Fecundity index: farrowing rate multiplied by average number of total piglets born per litter (total number of piglets born per 100 inseminations)

## Discussion

In the past few decades PCAI has emerged as one of the most successful reproductive biotechnology methods, and it is now used in a great number of countries [23]. However, its application is mostly limited to multiparous sows since its efficiency is reduced in primiparous [16] and nulliparous females [17]. In previous studies [18], it was demonstrated that the main problem using PCAI in nulliparous animals is crossing the cranial part of the cervix. In the present study a new device for depositing sperm deep in the gilt cervix is used, attaining a high level of successful application without impairing reproductive results and using a reduced sperm compared to that needed in traditional CAI.

The application success rate of the new insemination method (Dp-CAI) was close to 90%, which contrasts with the low level reached when PCAI is used in gilts, which ranges between 20% and 60% depending on the device and treatment used [17]. One of the main differences between the two techniques used in gilts (PCAI vs. Dp-CAI) is the length of the inner cannula, which in PCAI measures 16 cm from the cervical tip, compared with the 8 cm of Dp-CAI. These differences observed confirm that one of the main difficulties is to cross part of the cervix in nulliparous animals. In fact, other authors have reported that the introduction of the inner cannula beyond 10 cm of the cervix deposition only was possible in 44% of the gilts [6].

The morphology of the female genital tract varies with age and the number of parturitions [24–26]. Nulliparous females have a shorter cervix length (~ 4.3 cm) and cervical lumen diameter (~ 0.15 cm) compared to multiparous sows [18]. This may be one of the reasons for the low degree of successful application of PCAI in nulliparous compared with what is possible in multiparous animals. When the distance (cm) from the *rima vulvae* to the last cervical cushion was evaluated by our group [18] it was ~ 6 cm longer in multiparous sows. This, together with the fact that the average length of the Dp-CAI device remaining outside the gilt in this study was 27.23 cm, ensured that sperm deposition was intracervical.

The penetration length of the insemination device in gilts with two previous oestrus cycles was greater than in animals with only one cycle. The success rate of Dp-CAI tended to be greater when gilts had two previous oestrus cycles. The uterus varies in size in the transition from non-cyclic to cyclic gilts [27], thus facilitating the progression of the catheter during AI, which may explain the difference in penetration depth observed for the insemination device in the present study. Moreover, the age of the gilt influences the litter size as a possible consequence of an increased uterine capacity driven by oestrus-associated endocrine changes [28].

The extent to which the success of Dp-CAI was associated with the length of the insemination device remaining outside the gilt during AI (Fig. 2b) was also evaluated. As the length of the protruding AI device increased (i.e. the length of the device within the female genital tract decreased), the success rate of Dp-CAI decreased, reaching the lowest rate (less than 60%) when the protruding length was greater than 34 cm. These results suggest that the cervix impedes the progression of the catheter due to its reduced size impairing the introduction of the cannula. Although no correlation was observed between the length of the device outside the gilt and litter size (total and live piglets born), when the reproductive data were analysed by reference to catheter length sections (Fig. 3), the number of piglets born per litter was significantly reduced below a length of 34 cm, but there was no reduction in pregnancy rates. This reduction may be attributed to the low degree of development of the uterus in some gilts, which would affect its capacity to house the piglets. Nevertheless, there is a controversial association between insemination device penetration and litter size, some authors claiming a positive relationship [29] and others observing no relationship [28, 30].

One of the main goals that the swine industry hopes to achieve is a reduction in costs involved in pig production. One of the strategies to achieve this would be to decrease the number of spermatozoa inseminated per dose while maintaining the same reproductive efficiency [8]. Indeed, this is one of the reasons why PCAI has progressively displaced the traditional CAI. In the case of nulliparous gilts, PCAI cannot be applied with a high degree of confidence, and new alternatives have been developed [17, 19]. In this sense, Dp-CAI is presented as an alternative to PCAI and CAI for use in nulliparous animals. In our study, when nulliparous females were inseminated by Dp-CAI the resulting reproductive parameters were similar to those obtained with CAI but using 1 × 10^9^ fewer sperm cells per insemination (2 sessions of AI per oestrus cycle). This agrees with other reports that the deeper sperm can be deposited, the fewer sperm per AI dose are necessary without having a negative influence on the reproductive performance [7–9]. It has been demonstrated that the number of sperm reaching the oviduct is similar when using reduced sperm doses in both PCAI and CAI [31] and also the backflow (volume and number in terms of percentage from the initial seminal dose used) is reduced [8]. This indicates that the cervix acts as a serious barrier for sperm progression. In our case, this barrier could be partially bypassed by the cannula, further reducing the sperm dose when Dp-CAI is used.

One of the methodological differences between PCAI and Dp-CAI is the time necessary for sperm deposition. In contrast to the PCAI method, sperm deposition at the moment of insemination has to be performed slowly, more similarly to what is necessary with the CAI method. This is a consequence of not crossing the cervix barrier completely, and since the uterine part of the cervix is narrow more backflow can be observed if the insemination is performed quickly. The higher rate of backflow during insemination found in younger sows during CAI [32] is, probably due to the differences in size of the cervical canal. However, even though the Dp-CAI technique has to be performed slowly, the overall time consumed is less than in CAI due to the lower volume of seminal dose deposited (45 vs. 85 mL). Although the exact time required to carry out Dp-CAI was not evaluated in this study, it could be estimated (using 45 mL seminal doses) to be less than the 2.76 min (required for CAI method using a semen dose of 80 mL [8]) and more than the 1.12 min (required for the PCAI method using a 40 mL sperm dose [8]). So, the application of Dp-CAI in swine industry has similar advantages to those involved in PCAI including a reduction in the number of insemination doses per boar, reducing the number of boars needed per farm, and greater use of higher indexing boars among others (reviewed by [3]), thus ensuring substantial savings [8, 13]. However, it is important to note that an exhaustive economic study using this method in a large commercial production system and adapted to individual farm conditions still needs to be performed in situ in order to know the exact economic impact. Additionally, the 11% of animals which were not successfully inseminated using Dp-CAI may be a limitation for practical use in a large scale system, since CAI would still need to be used for those animals.

## Conclusions

The particular anatomy of the cervix in porcine females and the differences previously found in this anatomical part between nulliparous and multiparous animals have encouraged the development of new strategies and/or devices for deep AI in nulliparous females. The application of Dp-CAI using a new device specifically designed for gilts permits sperm to be deposited deep in the cervix with a high degree of success. Although additional studies are necessary into other aspects of Dp-CAI (lower sperm concentration doses, long term sperm conservation, genetic lines), this new method present itself as an alternative to PCAI and CAI in nulliparous females reducing the number of spermatozoa used per insemination without impairing the final reproductive performance, as demonstrated in a farm trial. The technique can be successfully used in gilts instead of the commonly used CAI method to reduce insemination costs.

## Additional files

Additional file 1: Reproductive parameters obtained after Dp-CAI (1.5 × 10^9^ sperm/45 mL) in gilts with 1 or 2 previously detected oestrus. Data show rate (%) or mean ± SD. No statistically significant differences were observed in any of the parameters studied (*P* > 0.05). (DOCX 19 kb)

Additional file 2: Association between length of device protruding from the gilt at insemination (Dp-CAI) with total a, and live b, piglets born. The relationship between length and litter size (total and live) was not significant (*P* > 0.05). (JPG 15000 kb)

## Abbreviations

AI: Artificial insemination
CAI: Cervical artificial insemination
DIUI: Deep intrauterine insemination
Dp-CAI: Deep cervical artificial insemination
PCAI: Post-cervical artificial insemination
