# Supplementary material for: A new device for deep cervical artificial insemination in gilts reduces the number of sperm per dose without impairing final reproductive performance
Source: J Anim Sci Biotechnol. 2019 Jan 28;10:11. doi: 10.1186/s40104-019-0313-1 (PMC6364433; doi:10.1186/s40104-019-0313-1)
Supplement: Supplementary file 1 — Reproductive parameters obtained after Dp-CAI (1.5 × 10^9^ sperm/45 mL) in gilts with 1 or 2 previously detected oestrus. Data show rate (%) or mean ± SD. No statistically significant differences were observed in any of the parameters studied (P > 0.05). (DOCX 19 kb) [file 40104_2019_313_MOESM1_ESM.docx]

**Additional file 1.** Reproductive parameters obtained after Dp-CAI (1.5×10^9^ sperm/45 mL) in gilts with 1 or 2 previously detected oestrus. Data show rate (%) or mean ± SD. No statistically significant differences were observed in any of the parameters studied (*P* > 0.05).

|  |  | **Number of gilts** | **Pregnancy,**  **%** | **Farrowing, %** | **Abortion, %** | **Total born per litter** | **Live born per litter** | **Fecundity index*** |
| --- | --- | --- | --- | --- | --- | --- | --- | --- |
| **Number of oestrus** | **1** | 102 | 88.9 | 85.9 | 3.0 | 12.7 ± 3.6 | 12.0 ± 3.5 | 1110.0 ± 320.4 |
|  | **2** | 934 | 89.9 | 87.7 | 2.2 | 13.1 ± 3.4 | 12.0 ± 3.5 | 1151.2 ± 303.5 |
|  | ***P*-value** |  | 0.429 | 0.346 | 0.396 | 0.289 | 0.484 | 0.289 |

*Fecundity index: farrowing rate multiplied by average number of total piglets born per litter (total number of piglets born per 100 inseminations).
