# Supplementary figures and images for: A new device for deep cervical artificial insemination in gilts reduces the number of sperm per dose without impairing final reproductive performance
Source: J Anim Sci Biotechnol. 2019 Jan 28;10:11. doi: 10.1186/s40104-019-0313-1 (PMC6364433; doi:10.1186/s40104-019-0313-1)

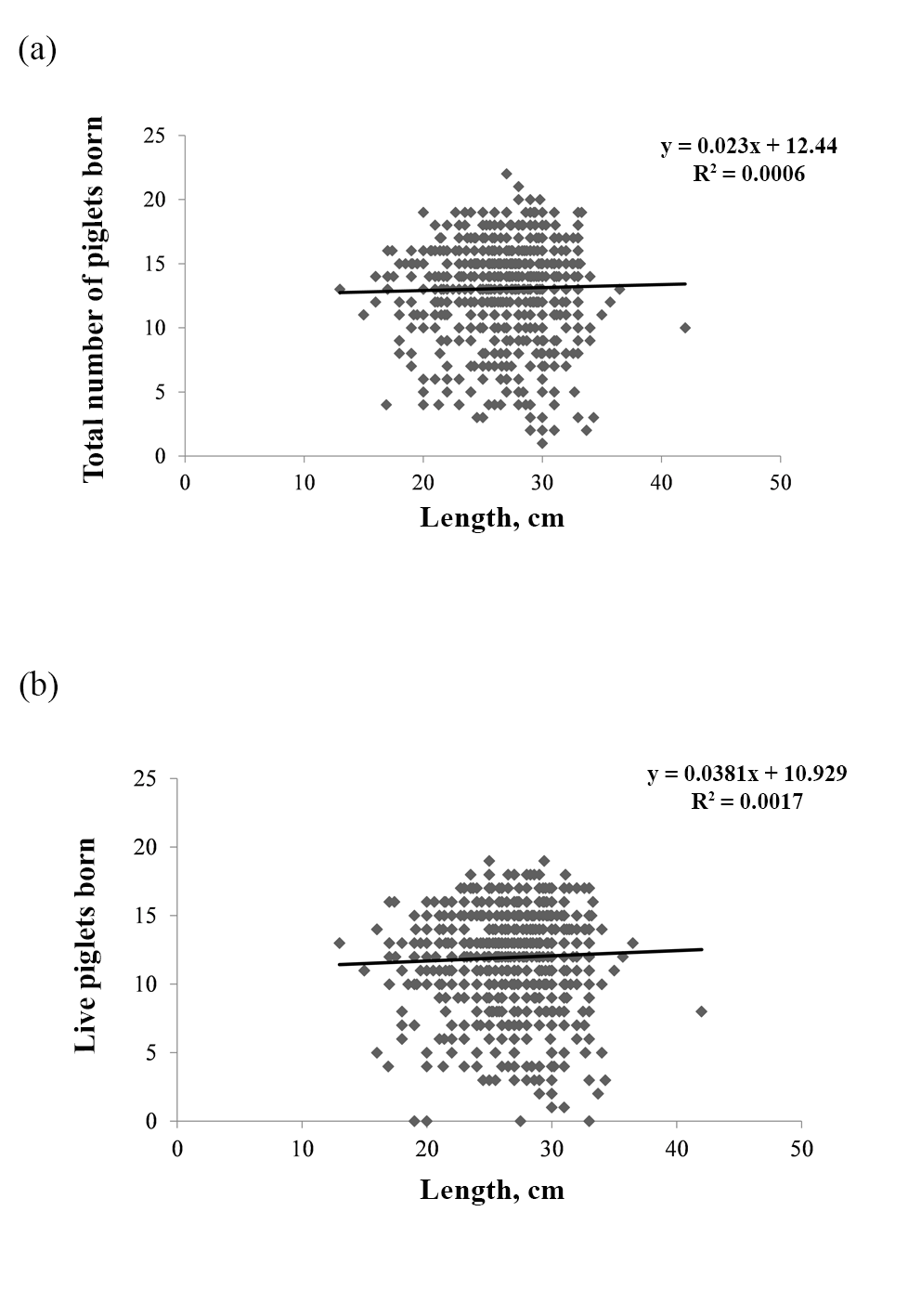

Supplement: Supplementary file 2 — Association between length of device protruding from the gilt at insemination (Dp-CAI) with total a, and live b, piglets born. The relationship between length and litter size (total and live) was not significant (P > 0.05). (JPG 15000 kb) [file 40104_2019_313_MOESM2_ESM.jpg]
